# COPD exacerbations in general practice: variability in oral prednisolone courses

**DOI:** 10.1186/1471-2296-13-3

**Published:** 2012-01-12

**Authors:** Marianne de Vries, Annette J Berendsen, Henk EP Bosveld, Huib AM Kerstjens, Thys van der Molen

**Affiliations:** 1 Department of General Practice, University Medical Centre Groningen, University of Groningen, Antonius Deusinglaan 1, FA 20, 9700 AD Groningen, the Netherlands; 2 Department of Pulmonology, University Medical Centre Groningen, University of Groningen, PO Box 30.001, 9700 RB Groningen, the Netherlands

## Abstract

**Background:**

The use of oral corticosteroids as treatment of COPD exacerbations in primary care is well established and evidence-based. However, the most appropriate dosage regimen has not been determined and remains controversial. Corticosteroid therapy is associated with a number of undesirable side effects, including hyperglycaemias, so differences in prescribing might be relevant. This study examines the differences between GPs in dosage and duration of prednisolone treatment in patients with a COPD exacerbation. It also investigates the number of general practitioners (GPs) who adjust their treatment according to the presence of diabetic co-morbidity.

**Methods:**

Cross-sectional study among 219 GPs and 25 GPs in training, located in the Northern part of the Netherlands.

**Results:**

The response rate was 69%. Nearly every GP prescribed a continuous dose of prednisolone 30 mg per day. Among GPs there were substantial differences in treatment duration. GPs prescribed courses of five, seven, ten, or fourteen days. A course of seven days was most common. The duration of treatment depended on exacerbation and disease severity. A course of five days was especially prescribed in case of a less severe exacerbation. In a more severe exacerbation duration of seven to fourteen days was more common. Hardly any GP adjusted treatment to the presence of diabetic co-morbidity.

**Conclusion:**

Under normal conditions GPs prescribe prednisolone quite uniformly, within the range of the current Dutch guidelines. There is insufficient guidance regarding how to adjust corticosteroid treatment to exacerbation severity, disease severity and the presence of diabetic co-morbidity. Under these circumstances, there is a substantial variation in treatment duration.

## Background

COPD exacerbations have a profound and long lasting effect on quality of life and the frequency of exacerbations contributes to long term decline in lung function [1]. Treatment of a COPD exacerbation with oral or parenteral corticosteroids significantly reduces treatment failure, the need for additional medical treatment, and shortens hospital stay. It increases the rate of improvement in lung function and dyspnoea [2]. However, corticosteroid therapy is associated with undesirable side effects, especially weight gain, insomnia and hyperglycaemias [2,3]. The risk of these side effects depends on dosage and duration [4].

The question is whether the potential benefits of treatment outweigh their risks. Optimum dosage and duration of treatment are not yet established [2]. Hospitalized patients all receive systemic treatment with corticosteroids. The Dutch College of General Practitioners (NHG) COPD Guideline recommends home treatment with prednisolone 30 mg for seven to fourteen days [5]. There is no guidance regarding how to adjust treatment to any co-morbidity.

Unclear treatment advice may lead to differences in dosage and duration of treatment with systemic corticosteroids. Because of the side effects, difference in prescribing might be relevant.

We addressed the following research questions:

Are there differences between general practitioners in the dose and duration of prednisolone treatment in case of COPD exacerbations?

Do dosage and duration of prednisolone treatment depend on exacerbation and disease severity or diabetic co-morbidity?

## Methods

A cross sectional survey using questionnaires was conducted in the Northern part of The Netherlands, distributed randomly over general practitioners (GPs) and GPs in training between May and June 2010.

The items of the questionnaire (additional file 1) were based on the literature and interviews with four experts. In order to improve the face and content validity, the concept questionnaire was presented to a number of senior GPs and GP trainees.

We presented four case scenarios regarding different patients with an exacerbation of COPD. In case A to D exacerbation-and disease severity increased.

Case A

A patient with a *mild to moderate *COPD (GOLD 1/2) and *no severe *exacerbation.

Case B

A patient with a *mild to moderate *COPD (GOLD 1/2) and a *severe *exacerbation.

Case C

A patient with a *severe to very severe *COPD (GOLD 3/4) and *no severe *exacerbation.

Case D

A patient with a *severe to very severe *COPD (GOLD 3/4) and a *severe *exacerbation.

An exacerbation was characterized by 'a worsening of patient's condition within a couple of days, representing a worsening of dyspnoea and coughing (with or without sputum) beyond normal day-to-day variations'. This definition is used in the COPD guideline of the Dutch College of General Practitioners (NHG). A severe exacerbation was characterized by dyspnoea at rest, inability to speak in a full sentence, not able to lay flat, respiratory frequency above thirty breaths per minute, heart rate above one hundred twenty beats per minute and the use of accessory respiratory muscles. The severity of COPD was defined as per GOLD classification (table 1). The questionnaire consisted of semi closed self-completed questions.

**Table 1:** Gold Classification: Global Initiative for Obstructive Lung Disease

	Severity	FEV1
**Gold 1**	Mild	≥ 80 predicted
**Gold 2**	Moderate	50-80% predicted
**Gold 3**	Severe	30-50% predicted
**Gold 4**	Very severe	≥ 30%, or < 50% predicted and with respiratory failure

Every GP had to give a treatment regimen for each case supposing that the patient was non-diabetic or diabetic, respectively. We choose the co-morbidity diabetes as this has the highest prevalence of the co-morbidities influenced by the use of prednisolone. Treatment regime was characterized by type of schedule (continuous/tapered), in combination with dosage (mg) and duration of treatment (days). We created space for comments.

Data were analysed by using SPSS 16.0 and described by percentages and confidence intervals. A p-value of < 0.05 was considered significant. Ethical approval was not required.

## Results

### Respondent characteristics

The study was conducted in May 2010 through to December 2010. Of the 240 included GPs 73% (n = 174) returned the questionnaire. A fully completed questionnaire was returned by 147 GPs and 19 GPs trainees (n = 166, 69%). All respondents were working in a general practice with an average of four days per week (range 0.5-5 days).

### Differences in dosage and duration of treatment with prednisolone

#### Dosage and duration of treatment

**Figure 1:**
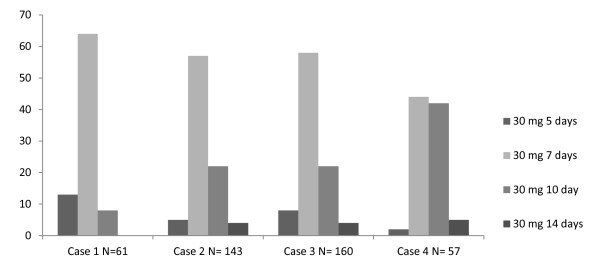
**Most common treatment regimens for COPD patients without diabetes**.

**Figure 2:**
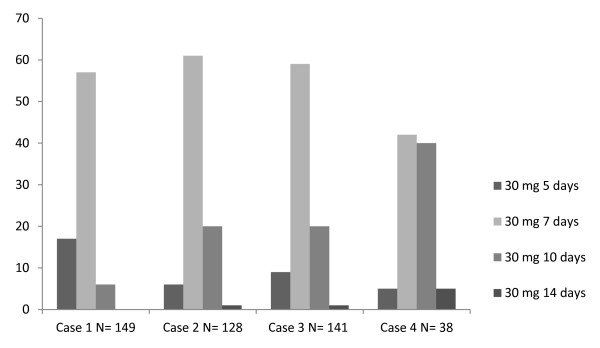
**Most common treatment regimens for COPD patients with diabetes**.

Nearly every GP gave a continuous regimen of prednisolone 30 mg a day for patients without or with diabetes (table 2 and 3; Figure 1 and 2). Among GPs there were large differences in duration of treatment. Although a seven- day course was most common, courses of five, ten, or fourteen days were also frequently prescribed in patients with or without diabetes. A five- day course was especially prescribed in case of a mild exacerbation. In more severe exacerbations, GPs preferred a course of seven, ten, or fourteen days. Only a few GPs prescribed the same regimen in each case (9%).

**Table 2:** Treatment of COPD exacerbations in general practice

	Case 1		Case 2		Case 3		Case 4	
	GOLD 1,2 no severe exacerbation		GOLD 1,2 no severe exacerbation		GOLD 3,4 severe exacerbation		GOLD 3,4 severe exacerbation	
	DM- [CI]	DM+ [CI]	DM- [CI]	DM+ [CI]	DM- [CI]	DM+ [CI]	DM- [CI]	DM+ [CI]
**Choice of treatment**	**N = 165**	**N = 161**	**N = 164**	**N = 159**	**N = 166**	**N = 158**	**N = 158**	**N = 156**
**1- **Specialist treatment	0% [0.000-0.000]	0% [0.000-0.000]	10% [0.057-0.154]	16% [0.104-0.223]	0% [0.000-0.000]	0% [0.000-0.000]	61% [0.527-0.684]	72% [0.640-0.787]
**2- **No treatment	2% [0.004-0.052]	5% [0.022-0.096]	0% [0.000-0.000]	0% [0.000-0.000]	0% [0.000-0.000]	1% [0.002-0.045]	0% [0.000-0.000]	0% [0.000-0.000]
**3- **Primary care treatment	98% [0.948-0.996]	95% [0.904-0.978]	90% [0.846-0.943]	84% [0.777-0.896]	100% [0.967-1.000]	99% [0.955-0.998]	39% [0.316-0.473]	28% [0.213-0.360]
**Type of regimen**	**N = 162**	**N = 157**	**N = 147**	**N = 134**	**N = 166**	**N = 157**	**N = 62**	**N = 43**
**A) **Tapering regimen	0% [0.000-0.000]	3% [0.010-0.073]	1% [0.000-0.037]	3% [0.008-0.075]	2% [0.004-0.052]	6% [0.027-0.106]	6% [0.018-0.157]	9% [0.026-0.221]
**B)** Continuous regimen	100% [0.966-1.000]	97% [0.927-0.990]	99% [0.963-1.000]	97% [0.925-0.992]	98% [0.948-0.996]	94% [0.894-0.973]	94% [0.843-0.982]	91% [0.779-0.974]
**Continuous regimen**	**N = 161**	**N = 149**	**N = 143**	**N = 128**	**N = 160**	**N = 141**	**N = 57**	**N = 38**
30mg for 5 days	13% [0.083-0.192]	17% [0.112-0.238]	5% [0.020-0.098]	6% [0.027-0.119]	8% [0.044-0.135]	9% [0.050-0.153]	2% [0.000-0.094]	5% [0.006-0.177]
30mg for 7 days	64% [0.560-0.714]	57% [0.487-0.651]	57% [0.488-0.656]	61% [0.519-0.694]	58% [0.501-0.659]	59% [0.503-0.671]	44% [0.307-0.576]	42% [0.263-0.592]
30mg for 10 days	8% [0.044-0.134]	6% [0.028-0.112]	22% [0.152-0.293]	20% [0.137-0.283]	22% [0.157-0.291]	20% [0.136-0.274]	42% [0.291-0.559]	40% [0.240-0.566]
30mg for 14 days	0% [0.000-0.000]	0% [0.000-0.000]	4% [0.016-0.089]	1% [0.000-0.043]	4% [0.014-0.080]	1% [0.000-0.039]	5% [0.011-0.146]	5% [0.006-0.177]

DM - = patients without diabetes; DM + = patients with diabetes; CI = 95% confidence interval

**Table 3:** Percentage of GPs adjusting their treatment in case of diabetic co-morbidity

		Case 1		Case 2		Case 3		Case 4	
		GOLD 1,2 no severe exacerbation		GOLD 1,2, severe exacerbation		GOLD 3,4, no severe exacerbation		GOLD 3,4, severe exacerbation	
	**Adjustment:**								
		**N = 162**	**CI**	**N = 157**	**CI**	**N = 153**	**CI**	**N = 154**	**CI**
1.	Higher total treatment dose	1%	[0.001-0.044]	1%	[0.002-0.045]	2%	[0.004-0.056]	0%	[0.000-0.000]
2.	Lower total treatment dose	11%	[0.067-0.170]	14%	[0.090-0.204]	14%	[0.087-0.202]	2%	[0.004-0.056]
3.	No treatment	3%	[0.010-0.071]	0%	[0.000-0.000]	1%	[0.002-0.046]	0%	[0.000-0.000]
4.	Specialist treatment	0%	[0.000-0.000]	5%	[0.022-0.098]	0%	[0.000-0.000]	10%	[0.056-0.156]
									
	**No adjustment:**								
5.	Both patients same total treatment dose	83%	[0.053- 0.148]	69%	[0.609-0.759]	83%	[0.761-0.886]	25%	[0.187-0.330]
6.	Both patients no treatment	2%	[0.004-0.053]	0%	[0.000-0.000]	0%	[0.000-0.000]	0%	[0.000-0.000]
7.	Both patients specialist line treatment	0%	[0.000-0.000]	11%	[0.064-0.168]	0%	[0.000-0.000]	63%	[0.548-0.706]

CI = 95% confidence interval

#### Dependency on exacerbation severity

Patients with a mild and a severe exacerbation were compared (case A to B and case C to D).

In patients with mild or moderate COPD, 46% of GPs took exacerbation severity into account. A majority of GPs gave a higher total dose of prednisolone in case of a severe exacerbation: on average, 88 mg prednisolone per treatment regimen more (35%, P < 0.001). Only 11% of patients received specialist treatment.

71% of GPs declared exacerbation severity to be an important factor in treating patient with severe or very severe COPD. In such cases, the majority of GPs preferred treatment by a pulmonologist (61%). A minority of patients was given a higher total dose of prednisolone: on average, 125 mg per regimen more (10%, P < 0.001).

#### Dependency on disease severity

Patients with GOLD 1/2 and GOLD 3/4 were compared (Case A to C and case B to D). 

In case of a mild exacerbation, 43% of GPs took into account disease severity by giving a higher total dose of prednisolone to patients with severe and very severe COPD: on average 116 mg prednisolone per treatment regime more (P < 0.001).

61% of GPs declared disease severity to be an important factor in treating patients with a severe exacerbation. Only a few GPs gave a higher total dose of prednisolone to patients with severe or very severe COPD. On average this was 118 mg more per treatment regime (10%, P < 0.001). Half of the GPs preferred treatment in a hospital (51%).

### Diabetes mellitus

Patients with and without diabetes mellitus were compared. Few GPs (16%) adjusted their treatment to the presence of diabetic co-morbidity (Table 3; Figure 2). In case of known diabetes, only 10% prescribed a lower total dose of prednisolone: case A to D on average respectively 73 mg (P < 0.001), 94 mg (P < 0.001), 87 mg (P < 0.001) and 66 mg (P < 0.002) prednisolone per treatment regime less. A few GPs gave no prednisolone treatment at all in case of a mild exacerbation (case A and C: 3% and 1%, respectively). Patients with a severe exacerbation were sometimes sent to hospital because of their diabetic co-morbidity (case B and D: 5% and 10%, respectively).

### Comments of GPs

The following factors where indicated to influence decisions on treatment regimen: individual treatment experience and the wish of the patient (n = 15), saturation (n = 8), home environment (n = 5), fever (n = 4), national guidelines (n = 2), advice pulmonologist (n = 2), the type of diabetes (n = 2), fluid intake (n = 1) and the continuous use of prednisolone (n = 1). Patients with diabetes administered a course of corticosteroids were advised to check their blood sugar more often (n = 26).

## Discussion

This study shows that GPs in the Netherlands rather uniformly prescribe a daily dose of 30 mg prednisolone in the treatment of COPD exacerbations. There is hardly any difference between GPs in dosage of treatment and with that they strictly seem to follow the guidelines. On the other hand GPs differed notably in duration of treatment. These differences in duration of treatment increased in more serious situations. A small number of GPs prescribed the same regimen in all case scenarios. Treatment was often adjusted to exacerbation and disease severity. This even determined the choice between treatment with or without consultation of a pulmonologist. Of note, few GPs adjusted their treatment to the presence of diabetic co-morbidity.

GPs reported the important influence of an earlier, successful individual treatment experience as well as the patients wish on choice of treatment regimen. It is conceivable that GPs management (working hours, local appointments, coded prescriptions and prescribing according to an electronic prescription system) as well as regional, national and international guidelines play a role in prescribing a particular treatment regimen.

Dutch GPs often follow the guidelines as issued by the NHG. These guidelines are evidence based, comprehensive, and the conclusions are published for all diseases in the same format. Currently guidelines are available for 99 different diseases among which COPD. The Second Dutch National study showed that GPs followed 76% of the recommendations [6]. Treatment advice was followed by 62% [6]. In our current study nearly every GPs declares to prescribe a continuous treatment regimen de facto according to the Dutch COPD guideline (96%). 80% of GPs followed the advised dosage and duration of treatment. The number of GPs practising according to the Dutch COPD guideline is therefore even higher than expected compared to the Second Dutch National Study.

The level of compliance with the guidelines is high even though there is hardly any evidence to support it. The regimen (30 mg prednisolone for 7-14 days) is also common internationally (table 4) [2,7-21]. A couple of studies comparing corticosteroid treatment with placebo treatment, showed a number of beneficial effects on FEV1 improvement, days of hospitalisation [7,8] and time to second exacerbation [9]. Only two studies were found regarding treatment duration [8,10]. In the first study, a course of 8 weeks was no more effective than a course of two weeks [8]. In the second study, a course of 10 days was more effective than a course of three days [10]. No head to head comparisons of different dosages were found. Randomised clinical trials (RCTs) using higher dosages did not achieve better results than RCTs using lower dosages [7-9]. The current policy to reject the tapering regimen is based on an old study in patients with an asthma exacerbation [16].

**Table 4:** National- and international guidelines: corticosteroid treatment in case of COPD exacerbation

Guideline	Recommendation
NHG- guideline COPD 2007	• Prednisolone 30 mg once a day for 7-14 days.
CBO guideline 2010: Diagnostics and treatment of COPD	• Prednisolone 30 mg once a day for 7-14 days. • Prefer oral prednisolone treatment. • Blood sugar check for patients with diabetes.
GOLD guideline 2009 'Global Strategy for the Diagnosis, Management, and Prevention of Chronic Obstructive Pulmonary Disease'	• Prednisolone 30-40 mg once a day for 7-10 days for patients with FEV1 < 50% • Budesonide, whether or not combined with formoterol may be an alternative to prednisolone treatment. • Prefer oral prednisolone treatment
ATS/ERS guideline 2004 'Standards for the diagnosis and treatment of patients with COPD'	• 30-40 mg prednisolone once a day for 10 days. • Consider corticosteroid inhalation therapy.
Nice guideline 2010: 'Chronic obstructive pulmonary disease: Management of chronic obstructive pulmonary disease in adults in primary and secondary care'.	• Prednisolone 30 mg once a day for 7-14 days.

It is interesting that few studies showed some beneficial effects of inhalation corticosteroids, whether or not combined with a long acting beta-2-agonist, in comparison with prednisolone [12-15].

With all these uncertainties it is not surprising that there is no guidance regarding how to adjust treatment to exacerbation and disease severity or to the presence of diabetic co-morbidity. Many GPs tend to prescribe a higher dose or longer treatment duration in case of a severe exacerbation or severe COPD. However again, evidence based regimes are missing. It is only known that oral prednisolone is not less effective than intravenous prednisolone [11].

Just like other international guidelines, the Dutch Guideline does not provide guidance how to prescribe in case of diabetic co-morbidity. However, dose and duration of treatment strongly determine the risk of side effects [4]. In nearly every patient with known diabetes, corticosteroids exacerbate hyperglycaemia [22]. Besides approximately 50% of patients without known diabetes, treated with 20 mg prednisolone a day, develop hyperglycaemia (> 11.1 mmol/l) within 24 hours [23]. So, many patients have to deal with steroid induced hyperglycaemia. This is not without risk. Fluctuations in plasma glucose concentrations have been associated with increased cardiovascular mortality [22]. Moreover, three or more corticosteroid treatments per year are associated with an odds ratio of 1.36 for the development of new onset diabetes [22].

The same treatment regimen was routinely applied to patients with or without diabetes mellitus. There is no evidence to support different doses, largely because there is no evidence at all. Perhaps clinicians feel that some loss of glucose control is not as serious as an inadequately treated exacerbation of COPD. Of the GPs 26 (16%) commented spontaneously that they advised their diabetic patients to check their blood sugar more often. An alternative for patients with diabetes could be to check their glucose levels daily preferably until a couple of days after finishing their treatment. It may be easier for patients on insulin to measure their blood sugar and adjust their insulin accordingly, while those on oral agents have less ability to do so.

Apart from hyperglycaemia there are important other side effects of the treatment with corticosteroids: in particular endocrine, neurological, psychiatric, ophthalmic and gastrointestinal side effects. There are also side effects of the musculoskeletal system, metabolism and electrolyte balance. Dosage and duration of treatment again determine the risk of these side effects [4]. For most of the side effects it is not exactly known at which dosage they occur. Side effects as fluid retention, hypertension and heart failure can occur directly after starting prednisolone treatment. Psychiatric disturbances as depression, mania, anxiety, and psychosis may occur within the first week [24]. Even short term, low dose systemic steroids exposes the patient to the risk of adrenal insufficiency [25]. A well-known long term complication is osteoporosis [26,27]. Many patients with COPD receive treatment with prednisolone several times a year. The cumulative dose of corticosteroids strongly correlates with vertebral fracture risk due to loss of bone mineral density [27].

Because of the short term side effects and the possible (cumulative) risks, total steroid exposition should be kept as low as possible. In patients with a COPD exacerbation, treatment should be as short as possible with the dosage prednisolone as low as possible. In patients with an asthma exacerbation a short course (3-5 days) of prednisolone has been shown to be effective [28,29].

More research should establish the optimum dose and duration of corticosteroid treatment. Research should also be directed at determining how to adjust corticosteroid treatment to exacerbation severity, disease severity and the presence of diabetic co-morbidity. Until then the best we can do is to follow the current guideline that recommends prednisolone 30 mg for seven to fourteen days.

Limitations of this study: Only GPs and GPs in training living in the North of Holland were approached. The response rate on the other hand was high (69%).

We did not want to increase the number of questions as this could influence the response rate. Therefore we decided to focus on dose and duration of prednisolone treatment, despite the recommendation of the Dutch guideline to double patient's dose of bronchodilators or to use a combination of two different bronchodilators in case of an non-severe exacerbation. Because diabetes is the most common co-morbidity, we only asked for an alternative regimen if the patient had diabetes. We did not make a difference for those on oral agents or on insulin. We did not focus on a possible concomitant therapy with antibiotics and we did not ask for the date of the last treated exacerbation. There may be a social desirability bias. However, the questionnaire does not concern a sensitive subject. Furthermore, assessing a patient on paper is not like assessing a patient in real life. It is likely that some GPs do not use a standard approach, resulting in less representative answers. We tried to solve this by offering an opportunity for comments.

## Conclusions

Under normal conditions GPs prescribe prednisolone quite uniform, within the range of the current guideline of the Dutch College of General Practitioners. There is insufficient guidance regarding how to adjust corticosteroid treatment to exacerbation severity, disease severity and the presence of diabetic co-morbidity. Under these circumstances, there is a substantial variation in treatment duration.

## Abbreviations

COPD: Chronic obstructive pulmonary disease; GP: General Practitioner; GOLD: Global Initiative for Obstructive Lung Disease; NHG: Dutch College of general practitioners; RCT: Randomised clinical trial.

## Competing interests

The authors declare that they have no competing interests.

## Authors' contributions

MV contributed substantially to the design and acquisition of data and drafted and edited the manuscript. AJB, HAM, and TM were involved in designing the study, supervising its execution and in drafting the manuscript as well as revising the manuscript critically. HEP carried out the data extraction. All authors read and approved the final manuscript.

## Pre-publication history

The pre-publication history for this paper can be accessed here:

http://www.biomedcentral.com/1471-2296/13/3/prepub

## Supplementary Material

Additional file 1 **The questionnaire - the items**. Click here for file
