# Supplementary material for: COPD exacerbations in general practice: variability in oral prednisolone courses
Source: BMC Fam Pract. 2012 Jan 12;13:3. doi: 10.1186/1471-2296-13-3 (PMC3323421; doi:10.1186/1471-2296-13-3)
Supplement: Additional file 1 — The questionnaire - the items. [file 1471-2296-13-3-S1.DOC]

**Questionnaire**

**COPD exacerbations in general practice: variability in oral prednisolone courses?**

1- Do you work in general practice?

- Yes, with an average of........days a week.
- No.

**Case 1**

**You decide to prescribe an oral course of prednisolone to a patient with mild to moderate COPD (GOLD 1, 2), no co-morbidity and no severe COPD exacerbation.**

2- What is your treatment regimen?

3- What is your treatment regimen in case of diabetic comorbidity?

- Continuous regimen of........mg a day for........days.
- Tapered regimen, starting with........mg a day for........days.
- Continuous regimen of........mg a day for........days.
- Tapered regimen, starting with........mg a day for........days.

**Case 2**

**You decide to prescribe an oral course of prednisolone to a patient with mild to moderate COPD (GOLD 1, 2), no co-morbidity and a severe COPD exacerbation.**

4- What is your treatment regimen?

- Continuous regimen of........mg a day for........days.
- Tapered regimen, starting with........mg a day for........days.
- Referral to specialist.

5- What is your treatment regimen in case of diabetic comorbidity?

- Continuous regimen of........mg a day for........days.
- Tapered regimen, starting with........mg a day for........days.
- Referral to specialist.

**Case 3**

**You decide to prescribe an oral course of prednisolone to a patient with severe to very severe COPD (GOLD 3, 4), no co-morbidity and no severe COPD exacerbation.**

6- What is your treatment regimen?

7- What is your treatment regimen in case of diabetic comorbidity?

- Continuous regimen of........mg a day for........days.
- Tapered regimen, starting with........mg a day for........days.
- Continuous regimen of........mg a day for........days.
- Tapered regimen, starting with........mg a day for........days.

**Casus 4**

**You decide to prescribe an oral course of prednisolone to a patient with severe to very severe COPD (GOLD 3, 4), no co-morbidity and a severe COPD exacerbation.**

8- What is your treatment regimen?

- Continuous regimen of........mg a day for........days.
- Tapered regimen, starting with........mg a day for........days.
- Referral to specialist.

9- What is your treatment regimen in case of diabetic comorbidity?

- Continuous regimen of........mg per a for........days.
- Tapered regimen, starting with........mg a day for........days.
- Referral to specialist.

Comments:

…………………………………………………………………………………………………………………………………………………………………………………………………………………………

………………………………………………………………………………………………………………………………………………………………………………………………………………………………………………………………………………………………………………………………………

*****Severe exacerbation : dyspnoea at rest, inability to speak a full sentence, not able to lie flat, respiratory frequency above thirty per minute, heart rate above one hundred twenty beats per minute and the use of accessory respiratory muscles.
